# KDGene: knowledge graph completion for disease gene prediction using interactional tensor decomposition

**DOI:** 10.1093/bib/bbae161

**Published:** 2024-04-11

**Authors:** Xinyan Wang, Kuo Yang, Ting Jia, Fanghui Gu, Chongyu Wang, Kuan Xu, Zixin Shu, Jianan Xia, Qiang Zhu, Xuezhong Zhou

**Keywords:** disease gene prediction, knowledge graph completion, tensor decomposition

## Abstract

The accurate identification of disease-associated genes is crucial for understanding the molecular mechanisms underlying various diseases. Most current methods focus on constructing biological networks and utilizing machine learning, particularly deep learning, to identify disease genes. However, these methods overlook complex relations among entities in biological knowledge graphs. Such information has been successfully applied in other areas of life science research, demonstrating their effectiveness. Knowledge graph embedding methods can learn the semantic information of different relations within the knowledge graphs. Nonetheless, the performance of existing representation learning techniques, when applied to domain-specific biological data, remains suboptimal. To solve these problems, we construct a biological knowledge graph centered on diseases and genes, and develop an end-to-end knowledge graph completion framework for disease gene prediction using interactional tensor decomposition named KDGene. KDGene incorporates an interaction module that bridges entity and relation embeddings within tensor decomposition, aiming to improve the representation of semantically similar concepts in specific domains and enhance the ability to accurately predict disease genes. Experimental results show that KDGene significantly outperforms state-of-the-art algorithms, whether existing disease gene prediction methods or knowledge graph embedding methods for general domains. Moreover, the comprehensive biological analysis of the predicted results further validates KDGene’s capability to accurately identify new candidate genes. This work proposes a scalable knowledge graph completion framework to identify disease candidate genes, from which the results are promising to provide valuable references for further wet experiments. Data and source codes are available at https://github.com/2020MEAI/KDGene.

## INTRODUCTION

Deciphering the molecular mechanisms underlying diseases is essential for the advancement of precision medicine [1]. One of the main goals is to identify the causing genes of diseases. Traditionally, this identification has relied heavily on experimental approaches, which are extremely time-consuming and labor-intensive [2].

With the completion of the Human Genome Project and the maturity of high-throughput sequencing technology [3], a growing body of computing-based Disease Gene Prediction (DGP) methods have been developed, which are proven effective [4, 5], mainly divided into four categories: (1) Network Propagation methods, which are mostly based on the classic random walk algorithm [6, 7]. (2) Methods based on Network Features. These methods usually use the constructed network to obtain the topological feature information of nodes, then calculate the correlation between a query disease and candidate genes, completing the prediction by sorting the gene list [8, 9]. (3) Supervised Learning methods such as classification [10]. And (4) Network Embedding and Deep Learning methods. These methods have gained wide attention in recent years [11, 12]. GLIM [13] can systematically mine the potential relationships between multilevel elements by embedding the features of the human multilevel network through contrastive learning. With the development of deep learning technology, researchers have tried to build specific neural network models to predict disease genes [14, 15]. Recent studies have further expanded the application of computational models in genomics, demonstrating innovative approaches for drug repurposing [16], predicting miRNA–disease associations [17] and exploring lncRNA–disease interactions [18], as well as employing hypergraph-based techniques for metabolite–disease interaction prediction [19]. These advancements highlight the continuous evolution of computational techniques in identifying disease-related genetic markers and potential therapeutic targets [20].

In recent years, Knowledge Graphs (KGs) have been successfully applied to life science research [21]. KG is a semantic network that reveals the relations between entities, which can formally describe things and their relations in the real world. In KGs, nodes represent entities or concepts, and edges are composed of attributes or relations. Knowledge exists in the form of triples [22]. Inferring unknown facts from those already in KGs is called Knowledge Graph Completion (KGC). Performing better in existing KGC models, Knowledge Graph Embedding (KGE)-based methods learn latent representations of entities and relations in continuous vector space [23]. One representative type of these methods is based on tensor decomposition. In Canonical Polyadic (CP) decomposition [24], a tensor can be decomposed into a set of matrices, where each row in the matrix represents an embedding vector of entity or relation. Since current implementations of CP are lagging behind their competitors, CP-N3 [25] uses a tensor nuclear p-norm as a regularizer to break through the limitations of CP and obtain considerable performance. Compared to KGE, Network Embedding (NE) assigns nodes in a network to low-dimensional representations and preserves the network structure effectively [26]. The main difference between KGE and NE is that the latter focuses on the topology of the network, while KGE focuses more on the internal information of different relations and the semantic connotation of facts.

At present, DGP methods combined with KGE have not been fully exploited. A few studies have explored KGE-based methods for disease gene prediction [27]. KGED [28] is a convolutional neural network-based KGE model, that uses external entity descriptions to infer relations between biological entities. Since KGED is used to predict gene-gene relations to generate gene interaction networks for diseases, it’s not an end-to-end model for DGP. And it requires textual descriptions of entities, which may introduce noise and are not simple to obtain. [29] and [30] adopt existing KGE models from the general domain. Although the conventional KGE models have been proven useful for inferring new biological relations, their performance with biological data is not as satisfactory as that of general-domain KGs [31]. One of the key points is how to model KGE in the process of disease gene prediction to accurately capture the interaction between biological entities [32], so that diseases and genes can be learned with more comprehensive biological features. Meanwhile, these researches present the KGE-based methods’ performance without comparison with existing DGP methods, which leaves the true performance of KGE still ambiguous.

**Figure 1:**
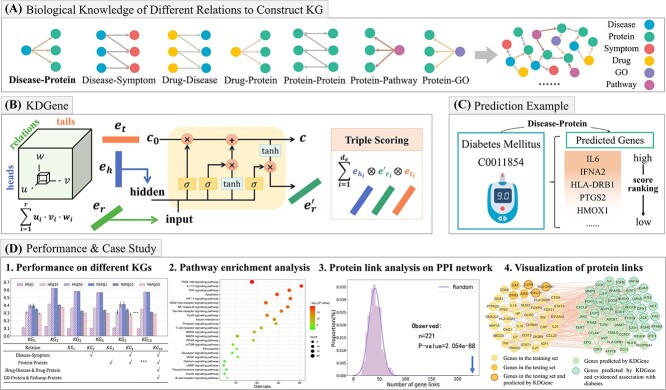
Visualization of our work. (**A**) We construct a comprehensive biological KG including seven relations. (**B**) After the construction, a triple is represented as $(h, r, t)$, with two entities $h, t$ and a relation $r$. We use $\boldsymbol{e_{h}}, \boldsymbol{e_{t}} \in \mathbb{R}^{d_{e}}$ to denote embeddings of head and tail entities and $\boldsymbol{e_{r}} \in \mathbb{R}^{d_{r}}$ to represent the relation embeddings. When embeddings $\boldsymbol{e_{h}}, \boldsymbol{e_{r}}, \boldsymbol{e_{t}}$ are trained, taking the relation embedding $\boldsymbol{e_{r}}$ as the input, the head entity embedding $\boldsymbol{e_{h}}$ as the hidden layer, we use the interaction module to obtain the updated relation embedding $\boldsymbol{e_{r}^{\prime}} \in \mathbb{R}^{d_{e}}$. Then the scoring function of triple $(h, r, t)$ is calculated by $\boldsymbol{e_{h}}$, $\boldsymbol{e_{r}^{\prime}}$ and $\boldsymbol{e_{t}}$. (**C**) After training, for a query disease, score all candidate genes and rank by descending as the prediction results. (**D**) Our framework’s performance, as well as case studies, demonstrates the model’s ability to identify accurate candidate genes.

To address these issues, we first integrated multiple relations centered on diseases and genes from biological knowledge bases to construct a large-scale biological KG, and develop an end-to-end knowledge graph completion framework using an interactional tensor decomposition to identify disease–gene associations, named KDGene (see Fig. 1A-D). KDGene introduces a gating mechanism-based interaction module between the embeddings of entities and relations to tensor decomposition, which can effectively enhance the information interaction in biological knowledge. Perceiving related knowledge, the model is capable of learning the connotation of different relations and endows biological entities and relations with more comprehensive and precise representations, which is beneficial to disease gene prediction. Experimental results show that KDGene performs optimally among existing DGP methods and conventional KGE methods. In particular, compared with KGE methods, KDGene realizes an average improvement of over 20% on Hit Ratio (HR) and Mean Average Precision (MAP) metrics. Moreover, KDGene’s performance in predicting unseen disease–gene associations further demonstrates its robustness and generalization capabilities. We also evaluate the impacts of KGs composed of knowledge with different relation types and degrees of confidence on its performance. In summary, the main contributions of our work are 3-fold:

1. We construct a biological knowledge graph centered on diseases and genes, then adopt a scalable end-to-end KGC framework to predict disease genes.
2. We propose a novel KGE model, called KDGene, specifically for disease gene prediction. The model introduces an interaction module to tensor decomposition, which effectively enhances the information interaction between biological knowledge.
3. The biological analysis, which includes case studies on diabetes mellitus and atrophic gastritis, also verifies KDGene’s capability to identify new and accurate candidate genes.

## MATERIALS AND METHODS

### Preliminaries

#### Knowledge graph

A knowledge graph can be denoted as $\mathcal{G} = \{ \mathcal{E}, \mathcal{R}, \mathcal{T} \}$, where $\mathcal{E}$ and $\mathcal{R}$ are the entity set and relation set, respectively. And $ \mathcal{T} = \{ (h, r, t) \in \mathcal{E} \times \mathcal{R} \times \mathcal{E} \}$ denotes the triple set which consists of all the triple facts in $\mathcal{G}$. When constructing a biological KG, we integrate knowledge from different biological databases in the form of triples and add them to the KG.

#### Knowledge graph completion

The task of KGC, also known as Link Prediction, is to either predict unseen relations *r* between two existing entities: (*h*, ?, *t*), or predict entities when the triple’s relation and another entity are given: (*h*, *r*, ?) or (?, *r*, *t*). For DGP, since triples of this kind of facts are in the form (disease, disease-gene, gene), we focus on the second mode to predict the tail entity (gene) given the head entity (disease) and relation (disease-gene).

In this paper, we adopt the improved tensor decomposition-based model under the framework of KGC, in which a triple (*h*, *r*, *t*) can be represented as an element of a binary third-order entity-relation tensor $\mathcal{X}^{\mathit{N} \times \mathit{M} \times \mathit{N}}$, where $\mathit{N}=|\mathcal{E}|$ is the total number of entities and $\mathit{M}=|\mathcal{R}|$ the number of relations. In the tensor $\mathcal{X}$, $\mathcal{X}_{ikj}$ denotes that there is a *k*th relation between the *i*th entity and the *j*th one, which is 


(1) \begin{align*}& \begin{aligned} \mathcal{X}_{i k j}= \begin{cases}1, & \text{ if}\ \left(h_{i}, r_{k}, t_{j}\right) \in \mathcal{G} \\ 0, & \text{ if}\ \left(h_{i}, r_{k}, t_{j}\right) \notin \mathcal{G}\end{cases} \end{aligned}\end{align*}

Therefore, tensor decomposition-based algorithms can infer a predicted tensor $\mathcal{\widehat{X}}$ that approximates $\mathcal{X}$. To predict the candidate genes of a disease, queries like (*i*, *k*, ?) are answered by ordering gene entities $j^{\prime}$ by decreasing scoring values of $\widehat{\mathcal{X}}_{i k j^{\prime}}$. Note that we propose a scalable KGC framework for disease gene prediction, which means the KGE model can be replaced by others.

### Biological KG construction

To learn more comprehensive representations of diseases and genes, we introduce the knowledge of different relation types to construct a biological KG(see Fig. 1A). Regarding diseases, the Disease–Symptom relations from SymMap [33] and the Drug–Disease relations from SIDER [34] are introduced into the KG. Regarding genes, we introduce the Protein–Protein Interactions (PPI) from STRING [35], the Drug-Protein relations from STITCH [36], the Protein–GO relations from GO [37] and the Protein–Pathway relations from KEGG [38]. Table 1 shows the scale of our biological KG.

**Table 1:** The scale of our constructed biological knowledge graph related to diseases and genes

Entity type	Quantity	Relation type	Quantity
Disease	22 697	Disease-Protein	117 738
Protein	21 616	Protein–Protein	841 068
Symptom	2504	Disease–Symptom	184 831
GO	1207	GO-Protein	61 634
Pathway	316	Pathway-Protein	25 813
Drug	1437	Drug–Disease	14 631
		Drug-Protein	277 745
Total	49 777	Total	1 523 460

In our framework, there are no restrictions on the entity type and relation type which means the construction of the KG is flexible. When others use it, the disease-gene relation facts can be added or subtracted from the KG to complete the training according to the demand. As the amount of knowledge in biological databases grows, abundant facts about new types of relations can be continuously added to our KG.

### CP-N3

The 3KGC task can be regarded as a 3D binary tensor completion problem, where each slice is the adjacency matrix of one relation type in the KG. It is a natural solution to apply tensor decomposition to the KGC task, which is simple, expressive and can achieve state-of-the-art results in general-domain KGs. Here, we take the typical CP-N3 model as an example and further introduce the interaction module on this basis to predict disease–gene associations.

CP-N3 [25] is based on CP decomposition [24], which decomposes a high-order tensor $\mathcal{X} \in \mathbb{R}^{n_{1} \times n_{2} \times n_{3}}$ into several $r$ rank one tensors $u_{i} \in \mathbb{R}^{n_{1}}, v_{i} \in \mathbb{R}^{n_{2}}, w_{i} \in \mathbb{R}^{n_{3}}$ ($\otimes $ denotes the tensor product): 


(2) \begin{align*}& \begin{aligned} \mathcal{X} \approx \sum_{i=1}^{r} u_{i} \otimes v_{i} \otimes w_{i}. \end{aligned}\end{align*}

### Interaction module

Introducing the interaction module aims to equip KGE models, tensor decomposition-based methods in particular, with better biological knowledge perception. That is, the model should learn more precise representations of entities and relations. To deal with the problem of long-term dependencies, Hochreiter and Schmidhuber proposed long short-term memory (LSTM) [39]. They improved the remembering capacity of the standard recurrent cell by introducing a gate’ into the cell in which the gate mechanism can choose which information enters the next cell [40]. We adopt the vanilla LSTM cell [41] consisting of an input gate, an output gate and a forget gate. The activation process of LSTM is as follows:

First, the forget gate $f$ and the input gate $i$ at the time step $t$ are computed by 


(3) \begin{align*}& \begin{aligned} f_{t} & =\sigma\left(W_{f h} h_{t-1}+W_{f x} x_{t}+b_{f}\right), \\ i_{t} & =\sigma\left(W_{i h} h_{t-1}+W_{i x} x_{t}+b_{i}\right), \\ \tilde{c}_{t} & =\tanh \left(W_{\tilde{c} h} h_{t-1}+W_{\tilde{c} x} x_{t}+b_{\tilde{c}}\right), \\ \end{aligned}\end{align*}

where $\sigma $ is the logistic sigmoid function. For the forget gate, the LSTM unit determines which information should be removed from its previous cell states $h_{t-1}$. The candidate memory cell $\tilde{c}_{t}$ is also added to the cell state through a TanH Layer. All the $W$ are weights that need to be learned, while $b$ represents the bias vector associated with this component. Then, the cell state is updated by 


(4) \begin{align*}& \begin{aligned} c_{t} & =f_{t} \circ c_{t-1}+i_{t} \circ \tilde{c}_{t}, \\ o_{t} & =\sigma\left(W_{o h} h_{t-1}+W_{o x} x_{t}+b_{o}\right), \\ h_{t} & =o_{t} \circ \tanh \left(c_{t}\right). \end{aligned}\end{align*}

where $o_{t}$ is the output gate, $c_{t}, h_{t}$ are the outputs at the current time and $\circ $ is the Hadamard product. In this intuitionistic structure, the control of the forget gate can save the previous information, and the control of the input gate can prevent the current irrelevant information from being added to the cell. The information in each part sufficiently interacts with others, which is why we utilize this simple and effective structure as our interaction module. By selecting this approach, we enable our model to better perceive and interpret the interactions between entities and relations, particularly within vertical biological domains.

### KDGene

We present KDGene (see Fig. 1B), a knowledge graph completion model that introduces the interaction module into CP-N3, which applies to disease gene prediction. In the following, a triple is represented as $(h, r, t)$, with two entities $h, t \in E$ (the set of entities) and a relation $r \in R$ (the set of relations). We use $\boldsymbol{e_{h}}, \boldsymbol{e_{t}} \in \mathbb{R}^{d_{e}}$ to denote the embeddings of head and tail entities and $\boldsymbol{e_{r}} \in \mathbb{R}^{d_{r}}$ to represent the relation embeddings.

Instead of adopting the translation-based principle $\boldsymbol{h} + \boldsymbol{r} = \boldsymbol{t}$ in TransE [42], we use the gating mechanism as the entity-to-relation translation. When the embeddings $\boldsymbol{e_{h}}, \boldsymbol{e_{r}}, \boldsymbol{e_{t}}$ are trained, taking the relation embedding $\boldsymbol{e_{r}}$ as the input, and the head entity embedding $\boldsymbol{e_{h}}$ as the hidden layer, we use an LSTM cell [39] to obtain the updated relation embedding $\boldsymbol{e_{r}^{\prime}} \in \mathbb{R}^{d_{e}}$. The calculation process is as follows: 


(5) \begin{align*}& \begin{aligned} f & =\sigma\left(W_{f h} \boldsymbol{e_{h}}+W_{f x} \boldsymbol{e_{r}}+b_{f}\right), \\ i & =\sigma\left(W_{i h} \boldsymbol{e_{h}}+W_{i x} \boldsymbol{e_{r}}+b_{i}\right), \\ \tilde{c} & =\tanh \left(W_{\tilde{c} h} \boldsymbol{e_{h}}+W_{\tilde{c} x} \boldsymbol{e_{r}}+b_{\tilde{c}}\right), \\ c & =f \circ c_{0} + i \circ \tilde{c}, \\ \boldsymbol{e_{r}^{\prime}} & =\sigma\left(W_{o h} \boldsymbol{e_{h}}+W_{o x} \boldsymbol{e_{r}}+b_{o}\right), \\ \end{aligned}\end{align*}

where all $W$ are weight matrices and $b$ are bias vectors learned in the training process. Similarly, $f,i, \tilde{c}, c$ are the forget gate, input gate, candidate memory cell and the call state, respectively. The initial input of the cell state is set to 0. The visual structure of the interaction module in KDGene can be found in Fig. S1 of the supplementary material.

After getting the updated relation embedding $\boldsymbol{e_{r}^{\prime}}$, we define the scoring function of a triple $(h, r, t)$ for KDGene as follows: 


(6) \begin{align*}& \begin{aligned} \phi(h, r, t) = \sum_{i=1}^{d_{e}} e_{hi} \otimes e_{ri}^{\prime} \otimes e_{ti}. \end{aligned}\end{align*}

In CP-N3, the embedding dimensions of entities and relations must be the same, resulting in a lot of parameter redundancy for those datasets with very different numbers of entities and relations. After introducing the interaction module, the dimensions of entities and relations can be different, which significantly improves the operability and flexibility of KDGene. More importantly, through the gating mechanism of LSTM, entities and relations are learned with more precise representations, which will benefit disease gene prediction.

### Training and prediction

We use the standard data augmentation techniques [25] of adding reciprocal predicates in the original training set $S$ and get $S^{\prime}$, i.e. add $(t, r^{-1}, h)$ for every $(h, r, t)$. Besides, we follow the 1-N scoring introduced by [43], that is, we take one $(h, r)$ pair and score it against all entities $t^{\prime} \in E$ simultaneously. We train our model with the full multiclass log-loss: 


(7) \begin{align*}& \begin{aligned} \mathcal{L} = \sum_{(h,r,t) \in S^{\prime}}\left( - \phi(h, r, t) + log\left(\sum_{t^{\prime} \in E}exp(\phi(h, r, t^{\prime}))\right)\right). \end{aligned}\end{align*}

where $\mathcal{L}$ is the loss function that should be minimized. For KDGene, we follow the N3 regularization used in CP-N3 [25], and the loss function for KDGene is as follows: 


(8) \begin{align*} \mathcal{L} = & \sum_{(h,r,t) \in S^{\prime}}\left( - \phi(h, r, t) + log\left(\sum_{t^{\prime} \in E}exp(\phi(h, r, t^{\prime})\right)\right) \nonumber \\ & + \lambda \sum_{i}^{d_{e}}(|e_{hi}|^{3} + |e_{ri}^{\prime}|^{3} + |e_{ti}|^{3})).\end{align*}

After training, for disease gene prediction, we take $(h,r)$ pairs, where the head entity is the query disease, and the relation is disease-gene, and then score all candidate genes that are not in the training set. The list of genes with high to low scores is the candidate genes’ prediction result (see Fig. 1C).

## EXPERIMENTAL RESULTS

### Experimental setting

#### Dataset

We select curated disease–gene associations from the DisGeNet database [44] as a benchmark dataset and apply the conventional 10-fold cross-validation to evaluate the disease gene prediction algorithms. For each fold, there are 117,738 disease–gene associations in the training set and 13,082 in the testing set.

#### Baselines

For baselines, comparisons with existing disease gene prediction algorithms are essential. Typical models including DADA [7], GUILD [45], RWRH [46], PDGNet [15], PRINCE [6], and RWR_PPI, RWR_HMLN, GLIM_DG [13] are our baselines. In addition, since we formulate disease gene prediction as the KGC task, and propose a novel KGE method, KDGene should also be compared with existing KGE models. We experiment with six popular KGE baselines: TransE [42], RotatE [47], DistMult [48], ComplEx [49], TuckER [50] and CP-N3 [25]. Open-source code links for these implementations can be found in our GitHub repository.

#### Evaluation metrics

Following [13], we select the hit ratio (HR@K) and mean average precision (MAP@K) as evaluation metrics (where K=1, 3, 10, 50). For a given disease $d$, $P_{d@K}$ represents the top-K predicted candidate genes, and $G_{d}$ represents the known genes of $d$ in the test set. $HR@K$ is calculated by 


(9) \begin{align*}& {HR@K}_{(d)}=\frac{\left|{P_{d@K}\bigcap G}_{d}\right|}{\left|G_{d}\right|}.\end{align*}

The Average Precision (AP) of a single disease $d$ can be calculated as follows: 


(10) \begin{align*}& {AP@K}_{\left(d\right)}=\frac{1}{\left|G_{d}\right|}\sum_{i\subseteq G_{d}}\frac{\sum_{j\subseteq G_{d}}{h\left(p_{j}<p_{i}\right)+1}}{p_{i}},\end{align*}

where $p_{i}$ is the position of candidate gene $i$ in the ranking list and $h\left (p_{j}<p_{i}\right )$ indicates that the position of candidate gene $j$ is higher than that of gene $i$ in the ranking list. The overall $MAP@K$ is the mean of $AP@K_{(d)}$ across all test diseases D: 


(11) \begin{align*}& MAP@K=\frac{\sum_{d}^{D}{AP@K}_{(d)}}{\left|D\right|}.\end{align*}

For both HR and MAP, higher values indicate higher predictive performance.

#### Implementation details

We implement KDGene with PyTorch and have made our source code and data available on https://github.com/2020MEAI/KDGene. In our experiments, we carried out extensive grid search, over the following ranges of hyperparameter values: batch size in {128, 256, 512, 1024}, learning rate in {0.01, 0.03, 0.05, 0.1}, regularization coefficient in {0, 0.001, 0.01, 0.1}, the entity and relation dimension in {1000, 1500, 2000}.

### Comparison with state-of-the-art models

Table 2 reports the evaluation results of disease gene prediction on the DisGeNet dataset. It can be seen that KDgene outperforms all the baselines consistently and significantly. Specifically, compared with DGP baselines, KDGene realizes an average improvement of 16.59% and over 25% improvement on the HR metric in particular. Compared with KGE-based baselines, in terms of HR@1, HR@3, HR@10, HR@50, MAP@1, MAP@3, MAP@10, MAP@50, the performance gains achieved by our model are 17.24, 21.44, 31.15, 20.35, 17.23, 21.12, 23.52 and 23.59%, with an average improvement of 21.96%. These results illustrate the effectiveness of KDGene for disease gene prediction.

**Table 2:** Disease gene prediction results on DisGeNet

	Models	HR@1	HR@3	HR@10	HR@50	MAP@1	MAP@3	MAP@10	MAP@50
DGP Methods	RWRH [46]	0.082	0.153	0.269	0.486	0.297	0.268	0.272	0.286
	PRINCE [6]	0.006	0.011	0.024	0.074	0.025	0.026	0.028	0.031
	DADA [7]	0.012	0.025	0.047	0.107	0.045	0.044	0.049	0.053
	GUILD [45]	0.023	0.032	0.049	0.107	0.073	0.076	0.080	0.084
	PDGNet [15]	0.020	0.031	0.045	0.068	0.094	0.056	0.044	0.043
	RWR_PPI [13]	0.070	0.148	0.271	0.474	0.257	0.241	0.255	0.270
	RWR_HMLN [13]	0.094	0.180	0.304	0.502	0.342	0.303	0.306	0.320
	GLIM_DG [13]	0.105	0.194	0.312	0.508	0.383	0.335	0.329	0.342
KGE Methods	TransE [42]	0.086	0.160	0.272	0.472	0.278	0.243	0.241	0.252
	RotatE [47]	0.085	0.159	0.272	0.477	0.275	0.241	0.241	0.252
	DistMult [48]	0.107	0.200	0.309	0.406	0.346	0.301	0.292	0.299
	ComplEx [49]	0.103	0.193	0.317	0.515	0.331	0.288	0.281	0.291
	TuckER [50]	0.096	0.182	0.288	0.394	0.308	0.269	0.261	0.269
	CP-N3 [25]	0.090	0.165	0.273	0.471	0.290	0.249	0.244	0.254
	**KDGene (ours)**	**0.126**	**0.243**	**0.416**	**0.620**	**0.406**	**0.365**	**0.361**	**0.370**

The table consists of two parts, the upper part is about the baselines for typical DGP models, and the lower is about the baselines for KGE models. **Bold** numbers are the best results of all and underline numbers are the best results of baseline models.

Our results also suggest that the success of KDGene does not mean that KGE-based models are the best in all DGP models (e.g. the MAP metric of GLIM_DG is better than all existing KGE methods), but they can still outperform most DGP models. It’s very promising for KGE-based methods to become the best in terms of the HR metric, showing a powerful recall ability, which indicates that combining complex relations in biological knowledge is beneficial for predicting candidate genes comprehensively.

Existing DGP models combined with KGE [29] usually adopt conventional KGE methods. Our experiments confirm that existing KGE-based models are effective but not necessarily optimal. Among these typical KGE models, methods based on tensor decomposition perform better (e.g. [48–50], CP-N3). Adopting tensor decomposition, we further introduce an interaction module based on the gating mechanism. It’s worth noting that the result of CP-N3 can be regarded as an ablation study. Compared with CP-N3, KDGene realizes an increase by 39.86, 47.35, 52.12, 31.81, 39.85, 46.30, 47.52 and 45.39% on HR@1, HR@3, HR@10, HR@50, MAP@1, MAP@3, MAP@10 and MAP@50, respectively, with an average improvement of 43.77%. The impressive improvement compared with CP-N3 demonstrates the significance of the interaction module, which enables KDGene with more precise representations of entities and relations to predict disease genes.

In addition to the model’s performance, we have also analyzed the computational cost and complexity of KDGene, with detailed discussions provided in Section III of the supplementary materials.

### Prediction on unseen disease–gene associations

We conduct a detailed evaluation of KDGene’s ability to predict gene associations for diseases not present in the training set. Specifically, we categorize the training data into two groups: Disease-gene in Train containing diseases with known gene associations in the training set, and Disease-gene not in Train containing diseases with no explicitly defined disease-gene relations in the training set but may present in the KG with other relations. A 10-fold cross-validation across DisGeNet is employed. On average, each fold featured 3409 diseases with known gene associations (Disease-gene in Train) and 321 diseases for which the model had not been directly exposed to the corresponding genes (Disease-gene not in Train). By adopting this approach, we aim to rigorously test KDGene’s generalization capabilities and its potential to uncover novel insights within the complex landscape of disease-gene relations. The results are presented in Table 3.

**Table 3:** Performance evaluation of models with/without prior exposure to disease–gene associations

Type	Models	HR@1	HR@10	MAP@1	MAP@10
Disease–gene in Train	CP-N3	0.090	0.273	0.290	0.244
	KDGene	0.126	0.416	0.406	0.361
	Improve	39.13%	52.23%	39.12%	46.92%
Disease–gene not in Train	CP-N3	0.028	0.129	0.029	0.054
	KDGene	0.065	0.186	0.067	0.099
	Improve	132.21%	44.87%	132.25%	82.75%

The results indicate that the prediction performance drops by nearly half when the model is not trained on the disease-gene triples, highlighting the huge challenge of no prior exposure. Despite this anticipated reduction, KDGene consistently surpasses the baseline CP-N3. Moreover, KDGene still marginally outperforms several extant disease gene prediction methods (i.e. PRINCE, DADA, GUILD, PDGNet) when Disease-gene not in Train. Our findings indicate that KDGene demonstrates promising performance in identifying potential disease–gene associations, even in the absence of direct training data for specific diseases. These results substantiate the utility of integrating biological knowledge graphs for augmenting the semantic richness of diseases and genes. They also underscore KDGene’s ability to leverage the comprehensive knowledge graph, effectively utilizing indirect information and relational patterns to predict associations.

### Comparison of different KGs

**Figure 2:**
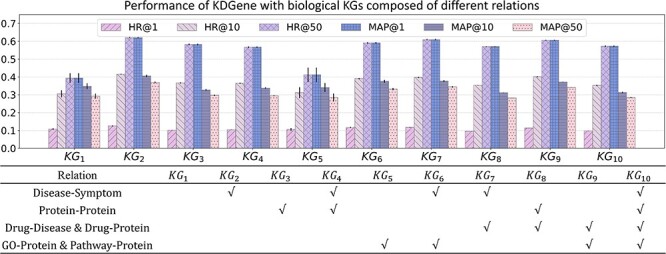
Results of KDGene with different biological KGs. We use seven relations associated with diseases and genes to evaluate the effect of ten combinations on performance. All these KGs include the disease-gene relation by default and other experimental settings remained constant.

To evaluate the impact of KGs composed of different relations on KDGene, we use different combinations of relations to construct biological KGs from $KG_{1}$ to $KG_{10}$ on KDGene. The results are shown in Fig. 2.

$KG_{1}$ consists only of disease-gene facts in the training set, with no external relations introduced. The corresponding overall performance is the worst. Based on $KG_{1}$, $KG_{2}$ and $KG_{3}$ introduce facts about Disease–Symptom and PPI, respectively. $KG_{2}$ achieves the best performance, indicating that disease–symptom associations are beneficial for candidate gene prediction, while PPI has little effect. $KG_{4}$, which jointly introduces the two relations, fails to achieve the cumulative effect.

Referring [30], $KG_{5}$ introduces GO and Pathway associations of genes. It can be seen from the results that only introducing protein-related facts does not improve the prediction of disease genes. $KG_{6}$ reintroduces the disease–symptom relation based on $KG_{5}$. And the performance has improved but still not reached the performance of $KG_{2}$. The possible reason for the degradation of $KG_{3}$ and $KG_{5}$ that further introduce Protein–Protein or GO & Pathway-Protein associations is that relations about proteins add more similar entities into the KG, which could be noise.

Within $KG_{7}$ to $KG_{10}$, these drug-related facts, when integrated into the biological KG, potentially enhance the model’s understanding of disease mechanisms by elucidating the therapeutic processes and the biochemical pathways involved in disease modulation. However, this improvement was modest, suggesting that while drug associations carry valuable signals, they may also introduce complexities or confounding factors that the model needs to disentangle. This trend is accompanied by a similar decrease in effectiveness when the KG is augmented with PPI relation ($KG_{8}$ and $KG_{10}$), thereby corroborating the principle that incorporating too many similar entities can detract from model performance.

### Comparison of different PPI score

**Figure 3:**
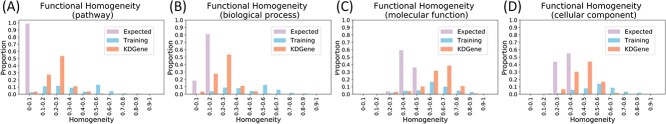
(**A**) Results of KDGene with facts in different confidence levels of protein-protein interactions. (**B**) Results of KDGene with different interaction modules.

To analyze the impact of knowledge with different confidence levels on KDGene, we consider scores in PPI facts. This score is often higher than the individual sub-scores, expressing increased confidence when an association is supported by several types of evidence [35]. We select three grades of scores for evaluation, that is, the interaction scores $\ge 700$, $\ge 850$ and $\ge 950$, respectively. For a fair comparison, all three evaluations are performed on the KG with the same disease-gene facts, with no differences other than the PPI facts. In Fig. 3(A), as the score threshold increases, the performance of KDGene gradually improves, which indicates the introduction of reliable biological knowledge into the KG is more beneficial for KDGene to learn the representations of entities and relations.

### Comparison of different interaction modules

To evaluate the impact of different interaction modules on the performance of KDGene, we conduct experiments with similar structures such as RNNCell and GRUCell, and the results are shown in Fig. 3(B). Among the three gating mechanisms, the relation embedding is used as the input, and the head entity embedding as the hidden layer. The results of introducing different interaction structures are all better than the model without the gating mechanism (here we compare with CP-N3), illustrating the significance of the interaction module for tensor decomposition models. Among the three, the result of LSTMCell is slightly better than the remaining two. The possible reason is that the setting of the forget gate makes it more parameters to learn more details.

### Hyper-parameter analysis of KDGene

We also conduct hyper-parameter tuning experiments focusing on embedding dimensions for entities and relations, batch size, learning rate, and regularization coefficient. Among these, the setting of the regularization coefficient has the most significant impact on KDGene’s performance, aligning with our baseline model CP-N3 [25] in general domain knowledge graphs. Notably, the introduction of the interaction module allows for the use of unequal embedding dimensions for entities and relations, enhancing flexibility beyond CP-N3. And the combination of differing dimensions has indeed shown better performance in our experiments. More detailed results can be found in Section II of the supplementary material.

### Functional homogeneity analysis

**Figure 4:**
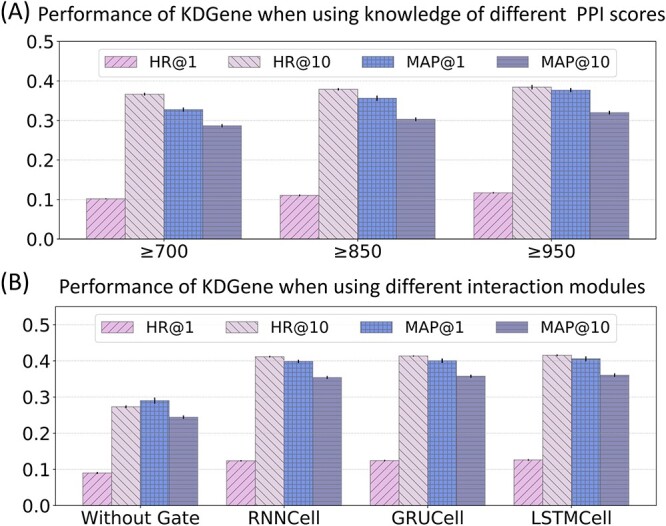
The result of functional homogeneity analysis of disease genes. (**A–D**) The distribution of the functional homogeneity of disease genes based on (A) pathways, (B) biological process, (C) molecular function and (D) cellular component of GO. The genes predicted by KDGene tend to be more functional related as compared with the random expectation.

To fully evaluate the candidate genes predicted by KDGene, we have conducted an in-depth functional homogeneity analysis [10, 51] for the candidate genes. First, we calculated the functional homogeneity [52] of the candidate genes (identified by KDGene) and the known genes of test diseases (named Training) and compared them with random expectation (named Expected). The results (see Fig. 4) showed that both candidate genes and known genes demonstrate a stronger functional homogeneity than that observed in the random experiment. The homogeneity of the genes predicted by KDGene proves to be even better than that of the known genes in molecular function, suggesting the biological relevance of the predicted candidate genes.

### Case study: diabetes mellitus and atrophic gastritis

**Figure 5:**
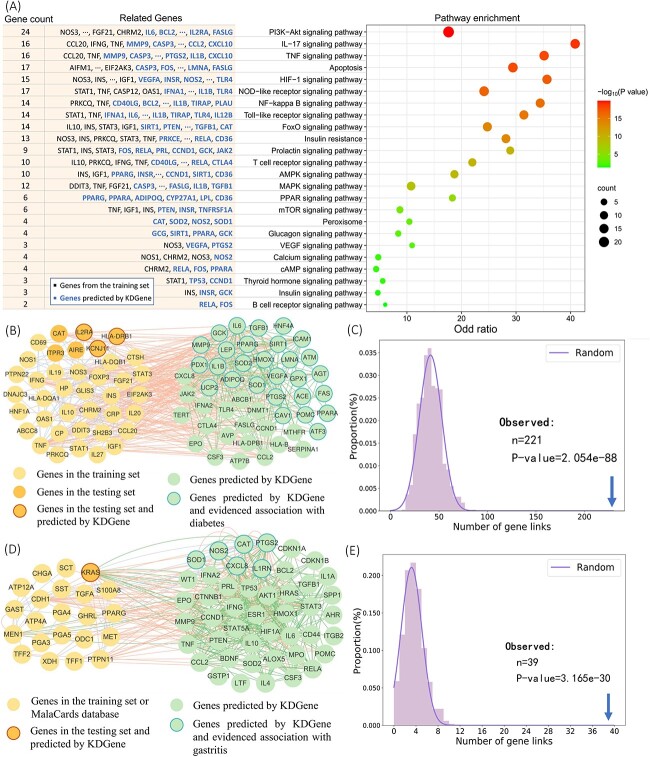
Case study. (**A**) Pathway enrichment analysis of the benchmark and candidate genes associated with diabetes mellitus. The bubble chart on the right illustrates significantly enriched pathways related to hypoglycemic pathways. The list on the left details the enriched genes, including those from the training set and new candidates predicted by KDGene. (**B**) Visualization of links of known and predicted genes for diabetes mellitus on the PPI network. (**C**) For diabetes mellitus, the observed number of network links is significantly larger than the random control (*P* = 2.05E-88, binomial test). (**D**) Visualization of links of known and predicted genes for Atrophic Gastritis on the PPI network. (**E**) The observed number of network links is significantly larger than the random control (*P* = 3.16E-30, binomial test). The statistical significance of the P-values for the predicted genes in both representative diseases suggests that the interactions observed on the PPI network are not due to random chance and may indicate biologically meaningful relationships relevant to the pathophysiology of the conditions.

We first perform pathway enrichment analysis on known genes in the training set and candidate genes predicted by KDGene for diabetes mellitus. The result is shown in Fig. 5(A). It can be seen that 24 pathways related to hypoglycemic pathways showed significant enrichment. Specifically, a significantly enriched pathway is the PI3K-Akt signaling pathway, including 24 related genes (5 from the training set and 19 predicted by KDGene), with a *P*-value of 1.25E-20. We also obtain the top 10predicted genes to demonstrate KDGene’s capability to seek out novel and credible candidate genes for diabetes mellitus (see Table 4). The independent database of disease-gene association (i.e. MalaCards) and literature database (i.e. PubMed) are utilized to validate the credibility of these candidate genes. We found that there are six candidate genes (precision=60%), namely IL6 (rank=1), HLA-DRB1 (rank=3), IL1B (rank=6), KCNJ11 (rank=7), IL2RA (rank=8), GCK (rank=9), that are recorded in MalaCards database. Especially, HLA-DRB1, KCNJ11 and IL2RA also exist in the testing set. Besides, all ten genes have co-occurrence with diabetes mellitus in current published literature, which declared these genes are likely to be associated with diabetes mellitus.

**Table 4:** Top 10 candidate genes for diabetes mellitus

Rank	Rank candidate gene	Recorded in MalaCards	Co-occurrence in related reference (PMID)
1	IL6	✓	16278864, 16150725
2	IFNA2		32160960, 34469050
3	HLA-DRB1	✓	10333055, 18279373
4	PTGS2		36049411, 33565572
5	HMOX1		34508760, 33343809
6	IL1B	✓	9112337, 11032727
7	KCNJ11	✓	32027066, 26448950
8	IL2RA	✓	28265534, 21248163
9	GCK	✓	9075802, 19933992
10	FAS		32168372, 30993294

Then, we take diabetes mellitus and atrophic gastritis as examples to illustrate the high network closeness and functional relevance between genes in the training set and the candidate genes predicted by KDGene (see Fig. 5). For diabetes mellitus (Fig. 5B–C), we keep all 42 genes in the training set and 6 genes in the testing set of the DisGeNet dataset and take out the top 50 candidate genes predicted by KDGene. The dense links (221 real links vs. 41.23 expected links, *P* = 2.05E-88, binomial test) that hold in the PPI network indicate that those two kinds of genes would tend to have closer interactions than expectation and rely on the same functional module in the PPI network. The top ten gene prediction results of KDGene hit three diabetes mellitus-associated genes in the testing set. Meanwhile, we find that more than half of the predicted genes have corresponding literature evidence for association with diabetes. For example, interleukin-6 (IL-6, the top predicted gene), is not in the testing set, but [53] indicates pro-inflammatory cytokines, such as interleukin-6 (IL-6), have been considered as key factors in type 1 diabetes mellitus and diabetic nephropathy.

For atrophic gastritis (Fig. 5D–E), due to the limited number of associated genes in the training and testing sets, we incorporate established disease–gene associations from the MalaCards database. This allows us to investigate the connectivity between genes with strong supporting evidence and the candidate genes predicted by KDGene in the PPI network. We detect 39 actual connections between predicted genes and evidence genes in the PPI network compared to the expected 3.192 connections (*P*=3.16E-30, binomial test), highlighting the closer and more relevant associations between predicted genes and evidence genes. Simultaneously, KDGene successfully predicts the only related gene, KRAS, in the testing set, and other genes in the top ten predictions, such as SOD2 and TNF, are validated as being associated with gastritis ([54, 55]). These results illustrate the accuracy and reliability of KDGene’s prediction, which are promising to provide valuable references for further wet experiments.

## DISCUSSION

In this study, we leverage biological knowledge bases to construct a KG and develop a scalable, end-to-end knowledge graph completion framework, KDGene, employing interactional tensor decomposition for identifying disease–gene associations. KDGene incorporates a gating mechanism-based interaction module between entity and relation embeddings, significantly enhancing biological knowledge learning. Our experimental findings establish KDGene’s superior performance over existing disease-gene prediction and knowledge graph embedding methods. Furthermore, we assess the effect of incorporating KGs with varying relation types and confidence levels on KDGene’s efficacy. The performance in predicting unseen disease–gene associations further demonstrates KDGene’s robustness and generalization ability, while comprehensive biological analyses on diseases like diabetes mellitus and atrophic gastritis validate its potential in identifying novel and accurate candidate genes.

However, despite comparing KDGene with current representative baselines in disease-gene prediction, the rapid development of computational biology databases, and the continuous emergence of related works pose challenges. These works, extending beyond disease-gene association prediction, employ the latest techniques like network embedding, deep learning and hypergraph-based approaches to address a wide spectrum of biological association predictions, including drug–disease [16], lncRNA–disease [18], metabolite–disease [19], miRNA–disease associations [17] and human–virus PPIs under different disease types [20]. Although our modeling approach shares similarities with these studies, differences in the data and feature level make it challenging to transfer these models for rigorous and fair comparison in disease-gene prediction performance.

In future work, we aim to incorporate the insights gained from these advanced methodologies, striving for continual optimization of our model to improve its predictive capabilities. By integrating the spirit underlying recent advancements in the field, we will enhance the accuracy and applicability of KDGene in the broader context of computational biology research.

**Key Points**

- We construct a biological knowledge graph centered on diseases and genes, then adopt a scalable end-to-end KGC framework to predict disease genes.
- We propose a novel KGE model, called KDGene, specifically for disease gene prediction. The model introduces an interaction module to tensor decomposition, which effectively enhances the information interaction between biological knowledge.
- The biological analysis, which includes case studies on diabetes mellitus and atrophic gastritis, also verifies KDGene’s capability to identify new and accurate candidate genes.

## Supplementary Material

KDGene-Supplementary_Material-240220-fina_bbae161

## Data Availability

The data and code of KDGene are available in https://github.com/2020MEAI/KDGene.

## Author Biographies

**Xinyan Wang** is a master student at the Beijing Key Lab of Traffic Data Analysis and Mining, School of Computer Science & Technology, Beijing Jiaotong University. Her research interests cover knowledge graph completion and artificial intelligence in medicine.

**Kuo Yang** is a lecturer at the Institute of Medical Intelligence, Beijing Key Lab of Traffic Data Analysis and Mining, School of Computer Science & Technology, Beijing Jiaotong University. His research interests cover network medicine and artificial intelligence in medicine.

**Ting Jia** received her MS degree in computer science from Beijing Jiaotong University. Her research interests include medical data mining.

**Fanghui Gu** is an associate researcher at the Key Laboratory of Carcinogenesis and Translational Research (Ministry of Education), Peking University Cancer Hospital & Institute. Her research interest lies in clinical oncology.

**Chongyu Wang** is a master student at the Beijing Key Lab of Traffic Data Analysis and Mining, School of Computer Science & Technology, Beijing Jiaotong University. His research interests include bioinformatics and artificial intelligence in medicine.

**Kuan Xu and Zixin Shu** are PhD students at the Beijing Key Lab of Traffic Data Analysis and Mining, School of Computer Science & Technology, Beijing Jiaotong University. Their research interests cover data mining and artificial intelligence in medicine.

**Jianan Xia** is a lecturer at the Institute of Medical Intelligence, Beijing Key Lab of Traffic Data Analysis and Mining, School of Computer Science & Technology, Beijing Jiaotong University. Her research interests include network medicine and artificial intelligence in medicine.

**Qiang Zhu** is an associate professor at the Institute of Medical Intelligence, Beijing Key Lab of Traffic Data Analysis and Mining, School of Computer Science & Technology, Beijing Jiaotong University. Her research interests include artificial intelligence in medicine.

**Xuezhong Zhou** is a professor at the Institute of Medical Intelligence, Beijing Key Lab of Traffic Data Analysis and Mining, School of Computer Science & Technology, Beijing Jiaotong University. His research interests cover network medicine, data mining and artificial intelligence in medicine.

## References

[1] Ashley EA . Towards precision medicine. Nat Rev Genet 2016;17(9):507–22. 27528417 10.1038/nrg.2016.86

[2] Calvo B , López-Bigas N, Furney SJ, et al. A partially supervised classification approach to dominant and recessive human disease gene prediction. Comput Methods Programs Biomed 2007;85(3):229–37. 17258838 10.1016/j.cmpb.2006.12.003

[3] Lander ES . Initial impact of the sequencing of the human genome. Nature 2011;470(7333):187–97. 21307931 10.1038/nature09792

[4] Zhou H , Skolnick J. A knowledge-based approach for predicting gene–disease associations. Bioinformatics 2016;32(18):2831–8. 27283949 10.1093/bioinformatics/btw358 PMC5018378

[5] Luo P , Chen B, Liao B, Fang-Xiang W. Predicting disease-associated genes: computational methods, databases, and evaluations. Wiley Interdiscip Rev Data Mining Knowl Discov 2021;11(2):e1383.

[6] Vanunu O , Magger O, Ruppin E, et al. Associating genes and protein complexes with disease via network propagation. PLoS Comput Biol 2010;6(1):e1000641. 20090828 10.1371/journal.pcbi.1000641 PMC2797085

[7] Erten S , Bebek G, Ewing RM, Koyutürk M. Da da: degree-aware algorithms for network-based disease gene prioritization. BioData Mining 2011;4(1):1–20. 21699738 10.1186/1756-0381-4-19 PMC3143097

[8] Xuebing W , Jiang R, Zhang MQ, Li S. Network-based global inference of human disease genes. Mol Syst Biol 2008;4(1):189. 18463613 10.1038/msb.2008.27 PMC2424293

[9] Jalilvand A , Akbari B, Mirakabad FZ, Ghaderi F. Disease gene prioritization using network topological analysis from a sequence based human functional linkage network. arXiv preprint arXiv:1904.06973. 2019.

[10] Yang K , Kezhi L, Yang W, et al. A network-based machine-learning framework to identify both functional modules and disease genes. Hum Genet 2021;140(6):897–913. 33409574 10.1007/s00439-020-02253-0

[11] Yang K , Wang N, Liu G, et al. Heterogeneous network embedding for identifying symptom candidate genes. J Am Med Inform Assoc 2018;25(11):1452–9. 30357378 10.1093/jamia/ocy117 PMC7646926

[12] Yang K , Wang R, Liu G, et al. HerGePred: heterogeneous network embedding representation for disease gene prediction. IEEE J Biomed Health Inform 2018;23(4):1805–15. 10.1109/JBHI.2018.2870728 31283472

[13] Hou S , Zhang P, Yang K, et al. Decoding multilevel relationships with the human tissue-cell-molecule network. Brief Bioinform 2022;23(5):bbac170. 10.1093/bib/bbac170 35551347

[14] Wu Y , Luo R, Leung H, et al. RENET: a deep learning approach for extracting gene-disease associations from literature. In: Cowen L. (ed) Research in Computational Molecular Biology. Lecture Notes in Computer Science, vol 11467. Springer, Cham, 2019, pp. 272–284.

[15] Yang K , Zheng Y, Kezhi L, et al. PDGNet: predicting disease genes using a deep neural network with multi-view features. IEEE/ACM Trans Comput Biol Bioinform 2020;19(1):575–584. 10.1109/TCBB.2020.3002771 32750864

[16] Fiscon G , Conte F, Farina L, Paci P. SaveRUNNER: a network-based algorithm for drug repurposing and its application to covid-19. PLoS Comput Biol 2021;17(2):e1008686. 10.1371/journal.pcbi.1008686 PMC7891752 33544720

[17] Ouyang D , Liang Y, Wang J, et al. Predicting multiple types of miRNA–disease associations using adaptive weighted nonnegative tensor factorization with self-paced learning and hypergraph regularization. Brief Bioinform 2022;23(6):bbac390. 10.1093/bib/bbac390 36168938

[18] Ma Y . DeepMNE: deep multi-network embedding for lncRNA-disease association prediction. IEEE J Biomed Health Inform 2022;26(7):3539–49. 35180094 10.1109/JBHI.2022.3152619

[19] Ma Y , Ma Y. Hypergraph-based logistic matrix factorization for metabolite–disease interaction prediction. Bioinformatics 2022;38(2):435–43. 34499104 10.1093/bioinformatics/btab652

[20] Ma Y , Zhong J. Logistic tensor decomposition with sparse subspace learning for prediction of multiple disease types of human–virus protein–protein interactions. Brief Bioinform 2023;24(1):bbac604. 36573486 10.1093/bib/bbac604

[21] Ye Q , Hsieh C-Y, Ziyi Yang Y, et al. A unified drug–target interaction prediction framework based on knowledge graph and recommendation system. Nat Commun 2021;12(1):6775. 34811351 10.1038/s41467-021-27137-3 PMC8635420

[22] Rossi A , Barbosa D, Firmani D, et al. Knowledge graph embedding for link prediction: a comparative analysis. ACM Trans Knowl Discov Data 2021;15(2):1–49.

[23] Wang Q , Mao Z, Wang B, Guo L. Knowledge graph embedding: a survey of approaches and applications. IEEE Trans Knowl Data Eng 2017;29(12):2724–43.

[24] Hitchcock FL . The expression of a tensor or a polyadic as a sum of products. J Math Phys 1927;6(1–4):164–89.

[25] Lacroix T , Usunier N, Obozinski G. Canonical tensor decomposition for knowledge base completion. In: International Conference on Machine Learning, vol. 80, Proceedings of Machine Learning Research, Stockholm, Sweden. PMLR, 2018, p. 2863–72.

[26] Grover A , Lescovec J. node2vec: Scalable feature learning for networks. In Proceedings of the 22nd ACM SIGKDD international conference on Knowledge discovery and data mining, San Francisco, California, USA. Association for Computing Machinery, 2016, pp. 855–864. 10.1145/2939672.2939754 PMC5108654 27853626

[27] Gao Z , Pan Y, Ding P, Rong X. A knowledge graph-based disease-gene prediction system using multi-relational graph convolution networks. In: AMIA Annual Symposium Proceedings, Vol. 2022. American Medical Informatics Association, 2022, p. 468–476. PMC10148306 37128437

[28] Choi W , Lee H. Identifying disease-gene associations using a convolutional neural network-based model by embedding a biological knowledge graph with entity descriptions. PloS One 2021;16(10):e0258626. 34653225 10.1371/journal.pone.0258626 PMC8519444

[29] Nunes S , Sousa RT, Pesquita C. Predicting gene-disease associations with knowledge graph embeddings over multiple ontologies. arXiv preprint arXiv:2105.04944. 2021.

[30] Vilela J , Asif M, Marques AR, et al. Biomedical knowledge graph embeddings for personalized medicine: predicting disease-gene associations. Exp Syst 2022;40:e13181.

[31] Choi W , Lee H. Inference of biomedical relations among chemicals, genes, diseases, and symptoms using knowledge representation learning. IEEE Access 2019;7:179373–84.

[32] Zhu C , Yang Z, Xia X, et al. Multimodal reasoning based on knowledge graph embedding for specific diseases. Bioinformatics 2022;38(8):2235–45. 35150235 10.1093/bioinformatics/btac085 PMC9004655

[33] Yang W , Zhang F, Yang K, et al. SymMap: an integrative database of traditional chinese medicine enhanced by symptom mapping. Nucleic Acids Res 2019;47(D1):D1110–7. 30380087 10.1093/nar/gky1021 PMC6323958

[34] Kuhn M , Letunic I, Jensen LJ, Bork P. The SIDER database of drugs and side effects. Nucleic Acids Res 2016;44(D1):D1075–9. 26481350 10.1093/nar/gkv1075 PMC4702794

[35] Von Mering C , Jensen LJ, Snel B, et al. STRING: known and predicted protein–protein associations, integrated and transferred across organisms. Nucleic Acids Res 2005;33(suppl_1):D433–7. 15608232 10.1093/nar/gki005 PMC539959

[36] Szklarczyk D , Santos A, Von Mering C, et al. STITCH5: augmenting protein–chemical interaction networks with tissue and affinity data. Nucleic Acids Res 2016;44(D1):D380–4. 26590256 10.1093/nar/gkv1277 PMC4702904

[37] The Gene Ontology Resource . Enriching a gold mine. Nucleic Acids Res 2021;49(D1):D325–34. 33290552 10.1093/nar/gkaa1113 PMC7779012

[38] Kanehisa M , Goto S. KEGG: Kyoto Encyclopedia of Genes and Genomes. Nucleic Acids Res 2000;28(1):27–30. 10592173 10.1093/nar/28.1.27 PMC102409

[39] Hochreiter S , Schmidhuber J. Long short-term memory. Neural Comput 1997;9(8):1735–80. 9377276 10.1162/neco.1997.9.8.1735

[40] Yong Y , Si X, Changhua H, Zhang J. A review of recurrent neural networks: LSTM cells and network architectures. Neural Comput 2019;31(7):1235–70. 31113301 10.1162/neco_a_01199

[41] Gers FA , Schmidhuber J, Cummins F. Learning to forget: continual prediction with LSTM. Neural Comput 2000;12(10):2451–71. 11032042 10.1162/089976600300015015

[42] Bordes A , Usunier N, Garcia-Duran A, et al. Translating embeddings for modeling multi-relational data. Adv Neural Inf Process Syst 2013;26:2787–95.

[43] Dettmers T , Minervini P, Stenetorp P, Riedel S, Riedel. Convolutional 2D knowledge graph embeddings. In: Proceedings of the AAAI Conference on Artificial Intelligence, vol. 32. Palo Alto, California, USA: AAAI Press; 2018; pp. 1811–18.

[44] Piñero J , Bravo À, Queralt-Rosinach N, et al. DisGeNET: a comprehensive platform integrating information on human disease-associated genes and variants. Nucleic Acids Res 2016;45:D833–9. 27924018 10.1093/nar/gkw943 PMC5210640

[45] Guney E , Oliva B. Exploiting protein-protein interaction networks for genome-wide disease-gene prioritization. PLOS ONE, 2012;7(9):1–12. 10.1371/journal.pone.0043557 PMC3448640 23028459

[46] Li Y , Patra JC. Genome-wide inferring gene–phenotype relationship by walking on the heterogeneous network. Bioinformatics 2010;26(9):1219–24. 20215462 10.1093/bioinformatics/btq108

[47] Sun Z , Deng Z-H, Nie J-Y, Tang J, Tang. RotatE: knowledge graph embedding by relational rotation in complex space. In: Proceedings of the International Conference on Learning Representations (ICLR), 2019.

[48] Yang B , Yih W-t, He X, et al. Embedding entities and relations for learning and inference in knowledge bases. In Proceedings of the International Conference on Learning Representations (ICLR), 2015.

[49] Trouillon T , Welbl J, Riedel S, et al. Complex embeddings for simple link prediction. In: Proceedings of The 33rd International Conference on Machine Learning, vol. 48, Proceedings of Machine Learning Research, New York, USA. PMLR, 2016, pp. 2071–2080.

[50] Balažević I , Allen C, Hospedales TM. TuckER: tensor factorization for knowledge graph completion. arXiv preprint arXiv:1901.09590. 2019.

[51] Wang Y , Yang K, Shu Z, et al. Network-based gene prediction for TCM symptoms. In: In 2020 IEEE International Conference on Bioinformatics and Biomedicine (BIBM), Seoul, Korea (South): IEEE, 2020, p. 2847–54.

[52] Liu G , Wang H, Chu H, et al. Functional diversity of topological modules in human protein-protein interaction networks. Sci Rep 2017;7(1):16199. 29170401 10.1038/s41598-017-16270-z PMC5701033

[53] Ururahy MAG , Costa KS, de Souza Y, et al. Association of polymorphisms in il6 gene promoter region with type 1 diabetes and increased albumin-to-creatinine ratio. Diabetes Metab Res Rev 2015;31(5):500–6. 25384728 10.1002/dmrr.2621

[54] Yi J-F , Li Y-M, Liu T, et al. Mn-SOD and CuZn-SOD polymorphisms and interactions with risk factors in gastric cancer. World J Gastroenterol 2010;16(37):4738–46. 20872977 10.3748/wjg.v16.i37.4738 PMC2951527

[55] Rahimian G , Abadi MSS, Mirzaei Y, et al. Relationship between mucosal TNF-$\alpha $ expression and Th1, Th17, Th22 and Treg responses in helicobacter pylori infection. AMB Express 2022;12(1):1–11. 36057049 10.1186/s13568-022-01456-0 PMC9440976

