# Supplementary material for: KDGene: knowledge graph completion for disease gene prediction using interactional tensor decomposition
Source: Brief Bioinform. 2024 Apr 11;25(3):bbae161. doi: 10.1093/bib/bbae161 (PMC11009469; doi:10.1093/bib/bbae161)
Supplement: KDGene-Supplementary_Material-240220-fina_bbae161 [file kdgene-supplementary_material-240220-fina_bbae161.pdf]

Supplementary Material

Section I. The visual structure of the interaction module in KDGene

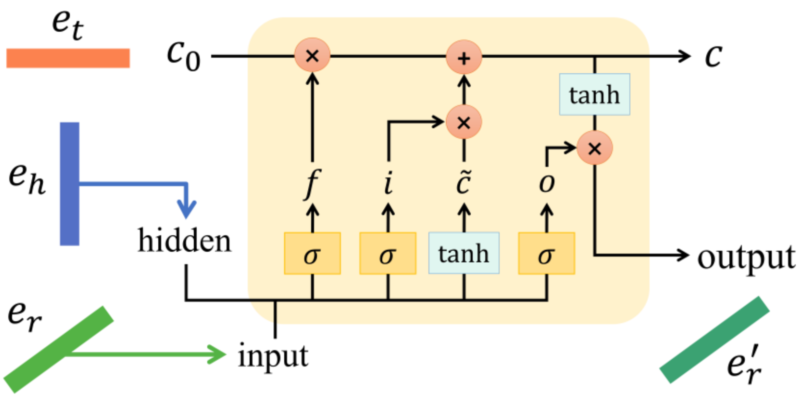

Supplementary Fig S1. Schematic Representation of the Interaction Module in KDGene. The diagram illustrates the LSTM-like architecture tailored for embedding entities e_h, e_t and relations e_r within the biological knowledge graph. The ‘input’ vector combines the embeddings of the head entity and relation, which are processed through a series of gates—'forget' (f), 'input' (i), and 'output' (o)—to regulate the flow of information. The 'hidden' state carries forward the processed information to the next time step. The 'cell state' (c) is updated by a combination of the previous cell state (c_0), the 'input' gate, and a 'candidate' memory ($\tilde{c}$) created by the 'tanh' function. The final output is produced after the 'output' gate modulates the cell state through a 'tanh' activation, symbolizing the updated relation embedding after the interaction.

Section II. Results of different hyper-parameter settings

The hyper-parameters of KDGene that demand our attention mainly include the embedding dimensions (comprising both entity and relation dimensions), batch size, learning rate, and regularization coefficient.

1) Embedding dimensions

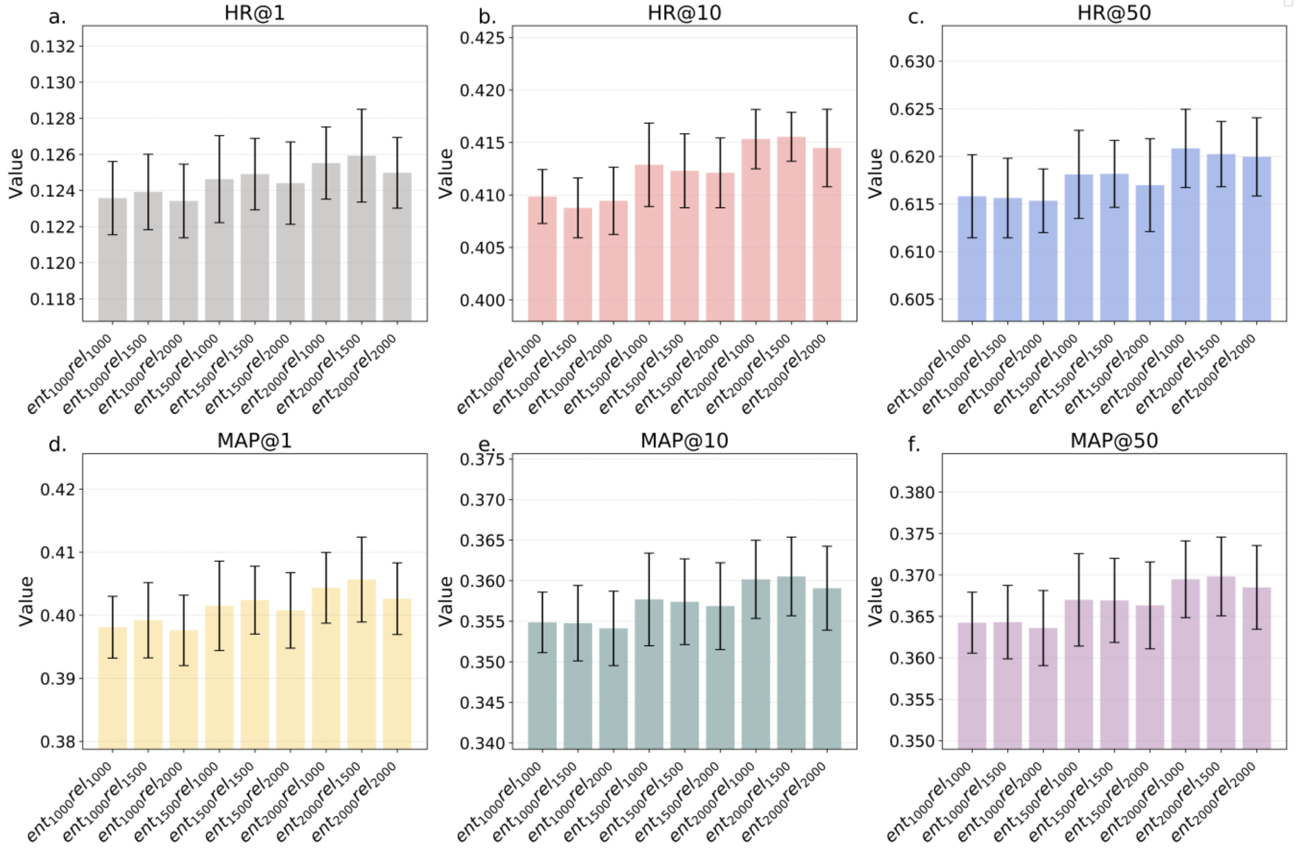

Supplementary Fig S2. Performance comparison of different entity and relation dimension combinations on model metrics. Subfigures (a), (b), and (c) show the Hit Ratio at 1, 10, and 50 (HR@1, HR@10, HR@50), respectively, while subfigures (d), (e), and (f) depict the Mean Average Precision at 1, 10, and 50 (MAP@1, MAP@10, MAP@50). Each bar represents the metric value for a specific combination of entity and relation dimensions, with error bars indicating the standard deviation across ten-fold cross-validation.

In determining the optimal embedding dimensions for entities and relations, we explored three values: 1000, 1500, and 2000, resulting in nine distinct combinations. Our analysis of these combinations revealed that the selection of 2000 dimensions for entities and 1500 for relations offered optimal performance. It is important to note, however, that the performance differences across various combinations were not substantial. This observation suggests that while our chosen configuration provides an effective balance, users may select alternative dimension settings based on the memory capacities of their systems. Such a decision allows for flexibility in resource allocation, ensuring that the model's deployment is efficient and practical.

2) Batch size

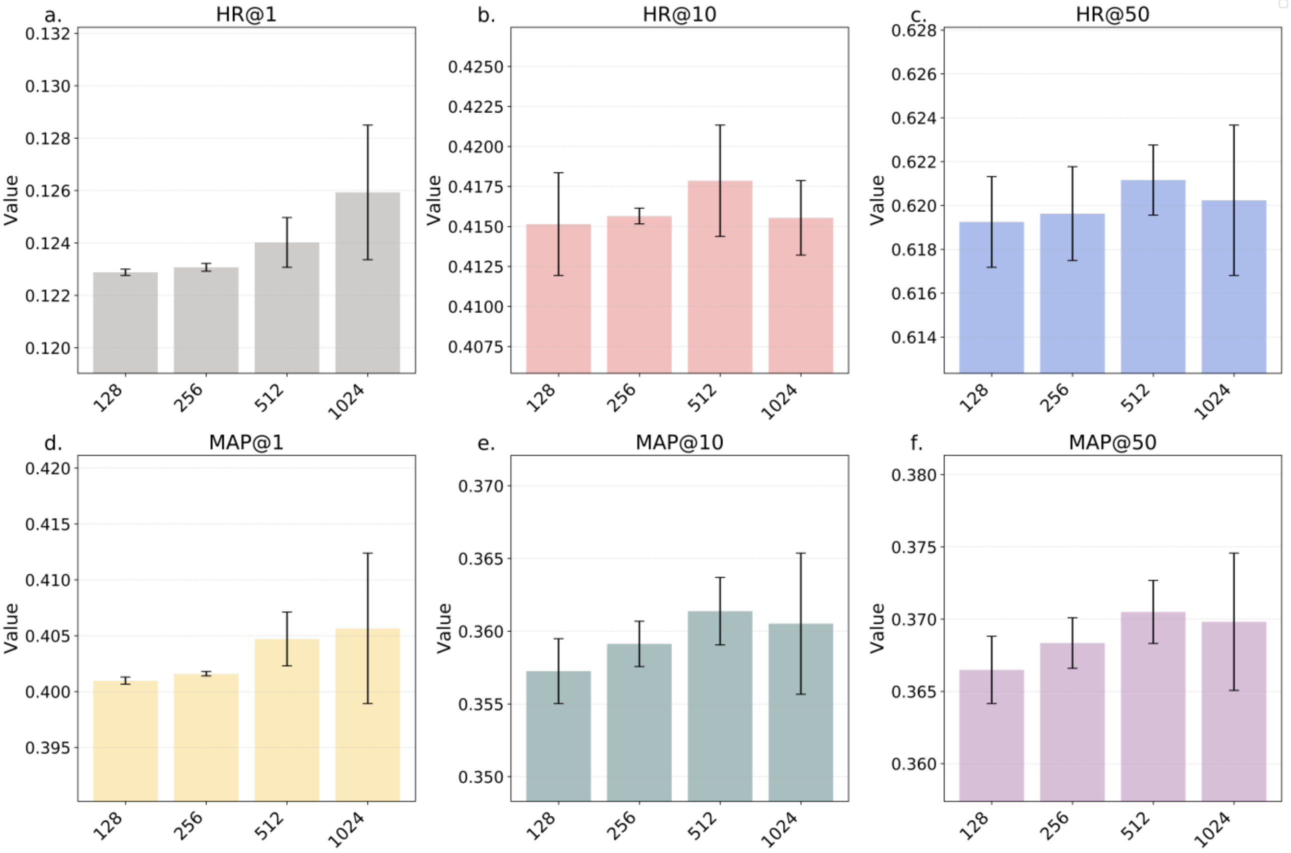

Supplementary Fig S3. Impact of batch size on model performance. Subfigures (a), (b), and (c) display the Hit Ratio at 1, 10, and 50 (HR@1, HR@10, HR@50) across different batch sizes, respectively. Subfigures (d), (e), and (f) present the Mean Average Precision at 1, 10, and 50 (MAP@1, MAP@10, MAP@50). Each bar corresponds to the metric value obtained with a specific batch size, where error bars denote the standard deviation observed over ten-fold cross-validation.

In our evaluation, we experimented with batch sizes of 128, 256, 512, and 1024. The aggregated results suggest that a batch size of 512 yields the best overall performance. However, practical considerations led us to adopt a batch size 1024 for our final model training. This decision was informed by recognizing that smaller batch sizes, while potentially more optimal, relatively increase training duration. By opting for the larger batch size, we struck a balance between computational efficiency and performance. Users are encouraged to adjust the batch size according to their specific computational constraints and training time considerations, thereby customizing the model training to their unique operational contexts.

3) Learning rate

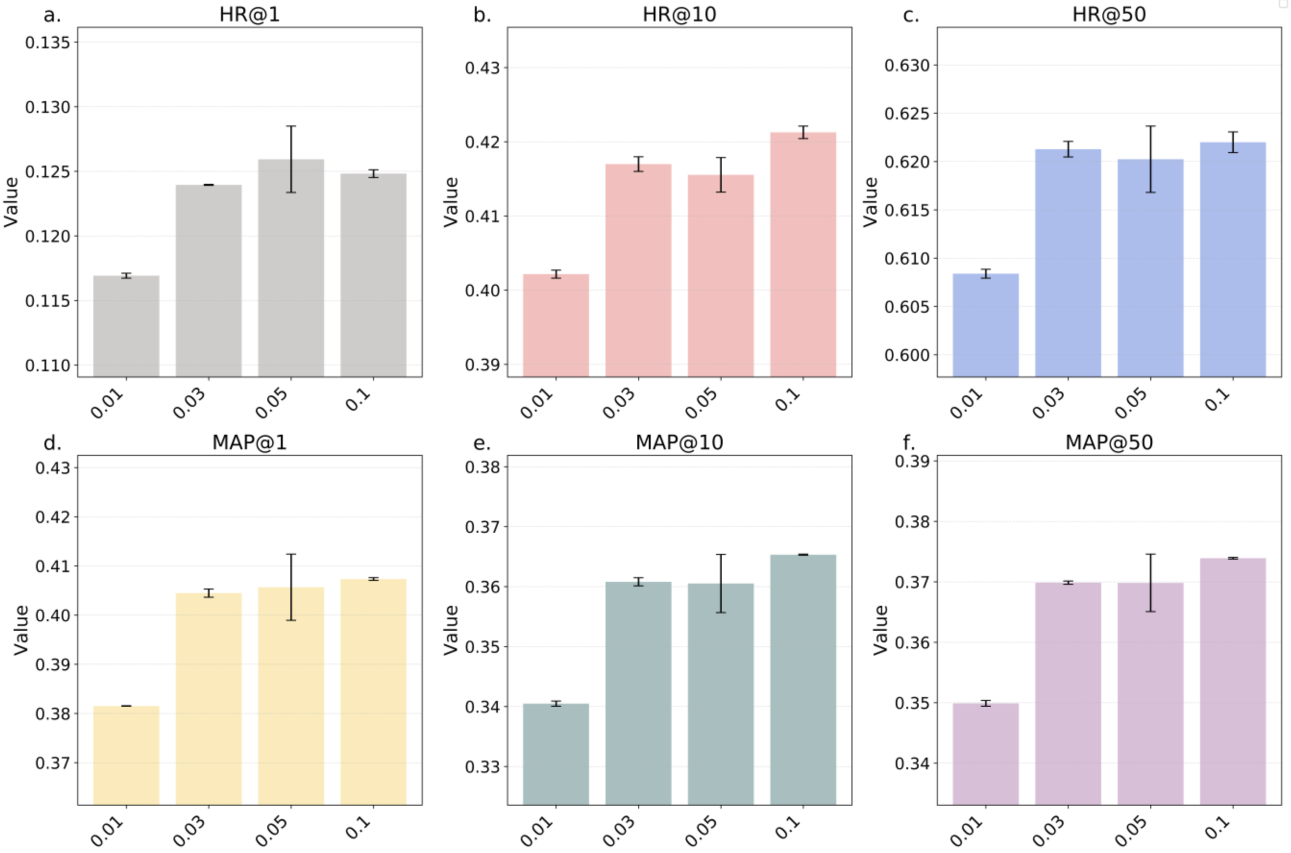

Supplementary Fig S4. Impact of learning rate on model performance. Subfigures (a), (b), and (c) display the Hit Ratio at 1, 10, and 50 (HR@1, HR@10, HR@50) across different learning rates, respectively. Subfigures (d), (e), and (f) present the Mean Average Precision at 1, 10, and 50 (MAP@1, MAP@10, MAP@50). Each bar corresponds to the metric value obtained with a specific learning rate, where error bars denote the standard deviation observed over ten-fold cross-validation.

When analyzing the effect of the learning rate on our model, we considered values of 0.01, 0.03, 0.05, and 0.1. We ultimately selected 0.05 for our model. Our selection of the 0.05 learning rate was based on its peak performance across our evaluations. However, the experimental results indicate that specifically for the DisGeNet dataset, a learning rate of 0.1 is also a feasible choice, providing satisfactory outcomes. It's important to consider that higher learning rates can lead to faster convergence but also carry the risk of missing the optimal solution. This factor becomes particularly crucial when adapting our model to various datasets, where the optimal learning rate may differ.

4) Regularization coefficient

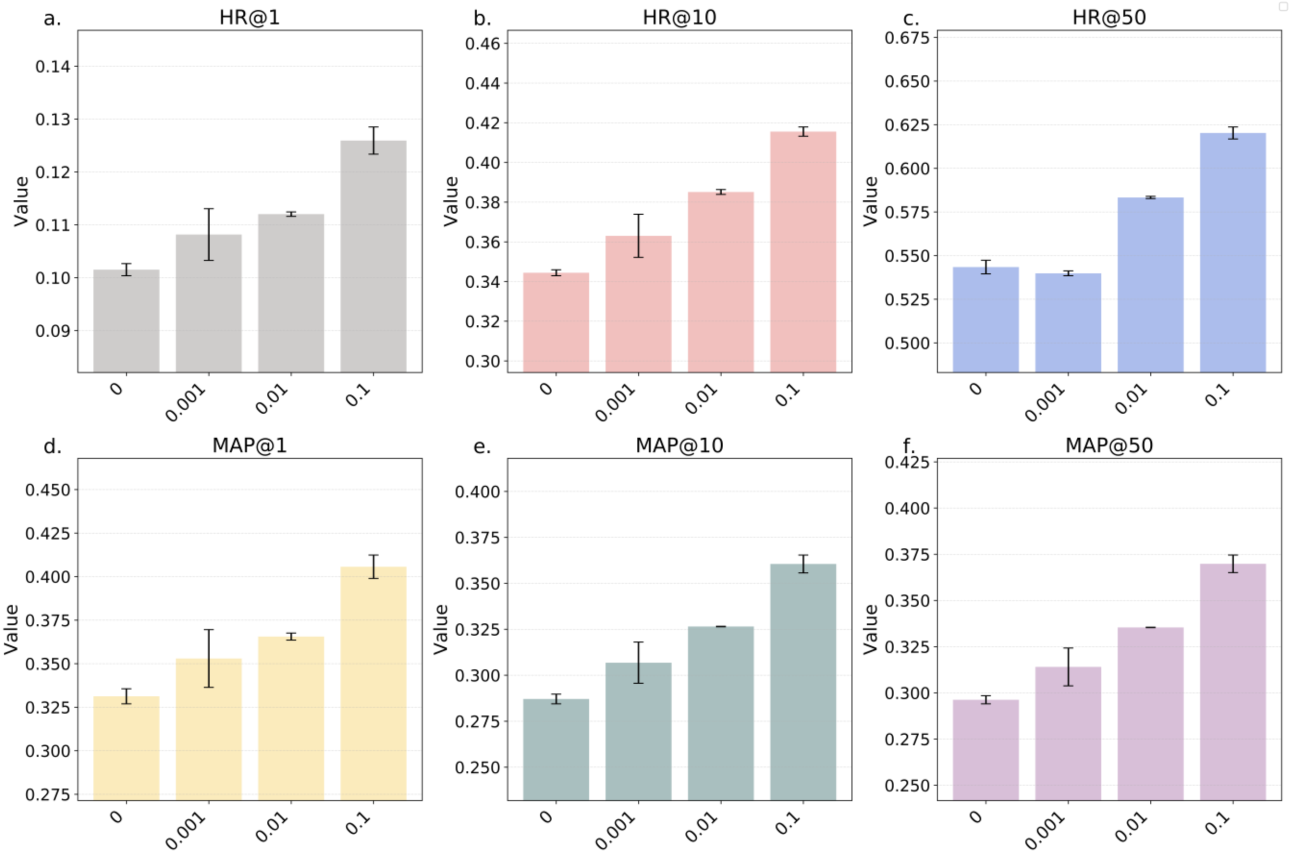

Supplementary Fig S5. Impact of regularization coefficient on model performance. Subfigures (a), (b), and (c) display the Hit Ratio at 1, 10, and 50 (HR@1, HR@10, HR@50) across different regularization coefficients, respectively. Subfigures (d), (e), and (f) present the Mean Average Precision at 1, 10, and 50 (MAP@1, MAP@10, MAP@50). Each bar corresponds to the metric value obtained with a specific regularization coefficient, where error bars denote the standard deviation observed over ten-fold cross-validation.

Our analysis for selecting the appropriate regularization coefficient considered values of 0, 0.001, 0.01, and 0.1. The performance metrics distinctly favored a coefficient of 0.1, indicating that this level of regularization effectively mitigates overfitting without overly constraining the model's capacity to learn from the data. A coefficient of 0, which corresponds to no regularization, typically leads to overfitting, as suggested by the lower performance metrics—a pattern that aligns with the outcomes observed for our baseline model CP-N3 [1] when applied to general knowledge graphs. This consistency in results reinforces the importance of regularization for tensor decomposition-based models, where the right amount of regularization is crucial for generalization and robust performance.

Section III. The computational cost and complexity of KDGene

We analyzed the computational cost and complexity of KDGene from the following three aspects: the scale of the knowledge graph, model parameters, and experimental details.

1) **The scale of the knowledge graph**

Our method introduces additional entities and relations, which naturally increases model parameters. This increase is scalable and depends on the size of the biological knowledge graph (KG). For example, incorporating the disease-symptom relationships into our KG significantly enhanced model performance with a marginal increase of 11,607 entities. In addition, each type of relation is represented by a 1500-dimensional vector (our current hyper-parameter setting). There are 7 types of relations in our KG now, which means 7 * 1500-dimensional vector storage is required. In other words, relational representation does not significantly increase memory overhead.

2) **Model parameters of KDGene**

The interaction module, unique to KDGene, is the recurrent neural network unit, which facilitates effective learning of representations in vertical-domain knowledge graphs through a straightforward yet powerful mechanism. This module in KDGene is implemented using a LSTMCell, which introduces additional parameters inherent to their design, including gates for regulating the flow of information (input, output, and forget gates) and cell states. Our selected hyper-parameters, with an entity dimension of 2000 and a relation dimension of 1500, reflect this design choice. While this increases the model's parameter count, it remains within manageable limits and can be adjusted based on computational resources available to the user. To assist researchers in optimizing these parameters, we have included a comprehensive analysis of hyper-parameter tuning in our supplementary material, thus enabling fine-tuning of the model's performance to fit computational constraints.

3) **Experimental details**

Our experiments were all conducted in PyTorch and on GeForce GTX 2080Ti GPUs. We have implemented optimization strategies, including efficient memory management and parallel processing, to keep the increased computational load manageable. The average training time was 86.75 seconds per epoch, with early stopping usually around 135 epochs. Comparatively, our baseline model CP-N3 recorded an average training time of 67.21 seconds per epoch across ten folds, with an average of 150 epochs until completion. Acknowledging the comparative analysis, it's evident that our model entails an average increase of 16.17% in training time compared to CP-N3. However, this investment in time is counterbalanced by a performance improvement of over 30%. This substantial enhancement in accuracy and predictive capabilities far outweighs the modest increase in computational effort.

References

[1] Lacroix T, Usunier N, Obozinski G. Canonical tensor decomposition for knowledge base completion[C]//International Conference on Machine Learning. PMLR, 2018: 2863-2872.
